# Docosahexaenoic acid in maternal and neonatal plasma phospholipids and milk lipids of Taiwanese women in Kinmen: fatty acid composition of maternal blood, neonatal blood and breast milk

**DOI:** 10.1186/1476-511X-12-27

**Published:** 2013-03-06

**Authors:** Hsiao-Ling Huang, Lu-Te Chuang, Hsi-Hsin Li, Chiu-Ping Lin, Robert H Glew

**Affiliations:** 1 Department of Healthcare Management, Yuanpei University, Hsinchu, Taiwan; 2 Department of Biotechnology, Yuanpei University, Hsinchu, Taiwan; 3 Department of Surgery, Kinmen Hospital, Kinmen, Taiwan; 4 Department of Gynecology, Kinmen Hospital, Kinmen, Taiwan; 5 Department of Surgery, School of Medicine, University of New Mexico, Albuquerque, NM, USA

**Keywords:** Breast milk, Lactation, Neonates, Fish intake, Kinmen, Docosahexaenoic acid, Pregnancy, Fatty acids

## Abstract

**Background:**

Docosahexaenoic acid (DHA) is a long-chain omega-3 polyunsaturated fatty acid (LCPUFA) that is critically important for the structure, development and function of the retina and central nervous system (CNS), ultimately contributing to improved cognition. It is known that the DHA content of breast milk is positively correlated with maternal DHA intake. Since there is a lack of information about the DHA status of pregnant and lactating women in rural Taiwan. The aims of the present study were to: 1) assess the DHA status of mothers and babies in urban setting, and 2) determine the content of DHA in the milk of nursing mothers.

**Methods:**

All pregnant women who attended the Obstetrics and Gynecology Outpatient Clinic of Kinmen Hospital on Kinmen Island in Taiwan between May 1 and May 30, 2011 were invited by research nurses to enroll in the study. The maternal blood sample was obtained on the day of their delivery. Cord blood was collected by the obstetrician following delivery. Participants were asked to visit the doctor forty-two days after the delivery, at which time a nurse collected breast milk on the day mothers were visiting the doctor for post-natal well-baby check-up.

**Results:**

The DHA percentages of maternal and neonatal plasma phospholipids were 5.16% and 6.36%, respectively, which are higher than values reported for most populations elsewhere in the world. The DHA percentage for the breast milk of Kinmen mothers was also high (0.98%) relation to international norms. The DHA proportions in maternal and neonatal plasma phospholipids were positively correlated (r = 0.46, p = 0.01).

**Conclusions:**

We show that the DHA status of mothers and newborns on Kinmen Island is satisfactory, thereby providing an evidence-based argument for promoting breastfeeding in Taiwan.

## Introduction

Docosahexaenoic acid (DHA) is a long-chain omega-3 polyunsaturated fatty acid (LCPUFA) that can be synthesized to only a limited extent in humans from the essential n-3 fatty acid α-linolenic acid (ALA). Thus, most of the human requirement for DHA must be satisfied by dietary means. It is widely acknowledged that DHA is a critically important nutrient for the normal development of infants. DHA plays essential roles in the structure and function of the retina and central nervous system (CNS) and it accumulates rapidly in these tissues both *in utero* and during the early stage of infancy [1-4]. Many years ago, Koletzko suggested that LCPUFAs with chain lengths of 20 to 22 carbon atoms play an essential role in the growth and maturation of the nervous system during infancy [5].

Bergmann and colleagues (2008) found that intake of 200 mg per day of DHA from mid-pregnancy through lactation is sufficient to support normal fetal neurodevelopment [6]. DHA also has a positive impact on the immunologic status of the newborn. Damsgaard and coworkers (2007) conducted a randomized control study and found that the ratio of the INF-γ/IL-10 cytokine profile of infant was higher in mothers who consumed fish oil or olive oil [7]. They concluded that a higher content of LCPUFA in breast milk was associated with enhanced the maturation of the infant’s immune system.

Numerous studies have shown positive relations between the blood DHA level and cognitive and visual function in breastfed infants [8-15]. DHA is present in breast milk where its concentration is largely dependent on the DHA intake of the mother [16,17].

In most parts of the world mothers are encouraged to breastfeed immediately following delivery and for at least six months thereafter [18,19]. Although recognition of the importance of the fatty acid composition of breast milk for an infant’s growth and development has stimulated extensive research work in many parts of world and even through public health officials in Taiwan have emphasized the benefits of breastfeeding and factors that promote breastfeeding [20-22], the literature contains few reports regarding the biochemical and nutritional aspects of breast milk.

Recognizing the unique geographical, historical and dietary habits of Taiwan, we sought to improve our better understanding of the nutrient content of breast milk among women living on Kinmen Island in order to advance prenatal care and public health programs for the mothers and babies living on this small island in the Taiwan Strait. To these ends, we determined the fatty acid composition of the phospholipids of maternal and infant (i.e., cord) plasma and the milk of Kinmen women. We also compared the DHA status of Kinmen women to that of women in other developed countries.

Specifically, the main aims of the present study were to: 1) assess the DHA status of mothers and babies in Kinmen, and 2) determine the content of DHA in the milk of nursing mothers. We hypothesized that since the population of Kinmen Island consumes large amounts of marine-food which is rich in DHA, mothers and newborns on the island should be well-nourished with regard to DHA and that nursing mothers should be producing milk that contains healthful amounts of DHA.

While it might seem intuitive that populations living close to the sea with ready access to seafood rich in DHA [23], this may not always be the case. For example, in a study conducted in Australia where the major population areas are located close to one ocean or another, it was found that only 6% of children met that nation’s suggested dietary target for long-chain LCPUFA [24] and that the overall intake of these fatty acids for all age categories of children was only 20% of the suggested dietary target (SDT).

## Methods

### Subjects

All pregnant women who attended the Obstetrics and Gynecology Outpatient Clinic of Kinmen Hospital on Kinmen Island in Taiwan between May 1 and May 30, 2011 were invited by research nurses to enroll in the study. The purpose and scope of the study was explained to them in writing, and the nurses were available to answer questions. Potential enrollees were ruled ineligible if they had preterm labor and delivery, preeclampsia, gestational or pre-gestational diabetes, other medical complications of pregnancy, or if their newborn weighed less than 2,500 grams. No prospective subject declined to participate and a total of 42 pregnant women were ultimately recruited into the study. Written informed consent was obtained from each participant. Subjects then completed a form with their contact information in order to allow a nurse to make arrangements for subsequent data collection. The maternal blood sample was obtained on the day of delivery. Cord blood was collected by the obstetrician following delivery. Participants were asked to visit the doctor 42 days after the delivery for the post-natal well-baby check-up, at which time a nurse collected breast milk. Blood samples (i.e., mother’s blood and cord blood) and breast milk were transported on crushed ice to the laboratory at Yuanpei University (Taiwan). The plasma fraction was collected and stored at -78°C. Ethical approval applicable to the present study under the Taiwanese ethical guidelines and which comply with the Declaration of Helsinki was obtained.

### Collection of dietary data of mothers

A validated food-frequency questionnaire (FFQ) [25-27] routinely employed in hospitals in Taiwan [28] was administrated to the pregnant subjects. Since the information derived from this FFQ reflects past nutrient intake rather than current dietary intake, it provided a means for obtaining information regarding food intake during pregnancy. There were 28 food items (e.g., fish, meat, vegetable, fruits) in the FFQ and weekly frequencies for consumption of these food items were recorded.

### Collection of blood samples

A venous blood sample was collected from each subject from the antecubital fossa into tubes containing ethylene diaminetetraacetic acid (EDTA) at the time of admission per standard labor and delivery protocol. Neonatal cord blood was collected after cord clamping as part of the routine delivery process. Within 72 h of delivery, nonhemolyzed maternal and neonatal samples were centrifuged for 5 min at top speed in a clinical centrifuge, and the plasma portion was removed and stored in 2-mL cryovials at -78°C until fatty acid analysis was performed within eight weeks.

### Collection of breast milk

Breast milk was collected after 42 days post-gestation to ensure that mature milk was fully established. With the aid of a manual pump, the first 3 mL of milk was collected and discarded. The next 10 to 15 mL of milk was collected into a sterile plastic cup and aliquoted into 2 mL cryovials.

### Collection of delivery-related data of mothers and infants

Information regarding mother’s age, height, weight, parity, gestational week, and mode of delivery was gathered. A mother’s weight was recorded one week before delivery. Neonatal data including gender, birth weight, height, and head circumstance were gathered from the delivery record.

### Chemicals

Triheptadecanoin were obtained from Sigma Chemical Co. (St. Louis, MO, USA). Gas chromatography (GC) and thin-layer chromatography (TLC) standard mixtures were purchased from Nu-Chek-Prep, Inc. (Elysian, MN, USA). All reagent-grade organic solvents were purchased from Burdick & Jackson (Muskegon, MI, USA).

### Fatty acid analysis

Total lipids from human plasma or milk were extracted according to the modified Folch method [29]. Briefly, 1 mL of plasma or milk sample was extracted with 20 mL of chloroform/methanol (2:1, v/v) at room temperature for 1 h. The total lipids in the organic phase were separated from the aqueous phase by adding 4 mL of saline solution (0.9% NaCl, w/v). After the chloroform phase was collected and evaporated to dryness at 40°C under nitrogen, and the total lipids were redissolved in 1 mL of chloroform. To separate the phospholipid fraction from total plasma lipids, 0.1 mL of the extract was then fractionated by thin-layer chromatography (TLC) using a developing solvent consisting of hexane/diethyl ether/acetic acid (80:20:1, v/v/v). The total phospholipid fraction was scraped from the plate, then reacted with 14% (w/v) boron trifluoride-methanol complex solution (BF_3_) (Sigma Chemical Co., St. Louis, MO, USA) at 95°C for 20 min to prepare fatty acid methyl esters (FAME). In the case of the milk samples, the total lipid fraction was subjected to fatty acid analysis.

The FAME of plasma phospholipids and milk fat were analyzed using an Agilent 6890 gas chromatograph equipped with a flame ionization detector, an antoinjector, a split/splitless injection system, and a fused-silica capillary column (Omegawax; 30 m × 0.32 mm, i.d., film thickness 0.25 μm, Supelco, Bellefonte, PA, USA). Helium was applied as the carrier gas. The temperature of the oven was programmed initially at 140°C, raised to 205°C at 6°C/min and held for 10 min. The temperatures of the injection port and detector were set at 205°C and 240°C, respectively. The FAME were identified by comparing their retention times to those of a known standard mixture RL-461 (Nu-Chek-Prep, Inc., Elysian, MN, USA). To quantify amounts of fatty acids, a known amount (16.6 μg) of triheptadecanoin (internal standard) was added to each lipid sample.

### Statistical analyses

Data gathered from the questionnaire and fatty acid analysis were coded into an Excel spreadsheet. Analysis of descriptive statistics and correlations was performed using SPSS 17.0 (SPSS, Inc., 2009, Chicago, IL, USA). The independent sample t-test was used to examine the mothers’ and the infants’ DHA levels. Correlations were performed between maternal characteristics and key fatty acids. The same statistical method, correlations, was conducted between maternal and neonatal plasma phospholipids and breast milk. A P value of 0.05 was considered significant.

## Results

### Characteristics of the study population

The characteristics of the mothers and their newborns are summarized in Table 1. A total of 42 mothers participated in the present study. The mean age of the mothers was 28.7 years and the average gestational week was 39.1. Means of mothers’ weight and height before the delivery were 62.1 kg and 160 cm, respectively. The average parity was 1.6 and all babies were delivered full-term. The majority of mothers (57%) had a vaginal delivery. For the neonates, the average weight, length and head circumstance were 3,153 g, 48.8 cm and 33.2 cm, respectively.

**Table 1:** Characteristics of mothers and their newborns

**Maternal (n = 42)**	**Mean (SD)**
Age (yrs)	28.7 (3.7)
Weight (kg)	62.1 (11.2)
Height (cm)	160 (44)
Gestational week	39.1 (1.2)
Parity	1.6 (0.8)
Neonate (n = 42)	
Weight (g)	3,153 (372)
Length (cm)	48.8 (3.5)
Head circumference (cm)	33.2 (2.8)

SD, Standard deviation.

With respect to seafood intake (Table 2), of women enrolled in the study, their average seafood intake was 1.6 meals per week. When we compared the amounts of food mothers consumed, they were more likely to consume meat, milk and vegetables (more than seven meals per week) than fruits, eggs and seafood (less than seven meals per week).

**Table 2:** Food intakes of mothers

**Food item**	**Consumption frequency (meals per week)**
	Mean (SD)
Seafood	1.6 (1.0)
Meat	8.4 (4.5)
Milk	8.3 (6.6)
Vegetable	7.2 (4.4)
Fruit	6.8 (4.6)
Egg	5.0 (2.7)

### Fatty acid composition of milk lipids

The main reason for conducting this study was to compare the DHA percentage in the milk fat of women in Kinman to the proportion of DHA that is widely regarded as necessary for meeting the DHA needs of the exclusively breast-fed infant during the first six months of life (e.g. 0.3 - 0.4%) [30]. The mean DHA percentage for the milk of the Kinman women was 0.98% (Table 3). The proportion of eicosapentaenoic (EPA), another n-3 long-chain fatty acid considered to be critical for normal development of the central nervous system, was about one-fifth that of DHA but well within the range of values for lactating women in most other developed countries. The essential fatty acids linoleic acid (LA) and α-linolenic acid (ALA) accounted for 22.8% and 1.46%, respectively, of the fatty acid total in the milk fat. Arachidonic acid (AA) accounted for 0.93% of the fatty acid total. The monounsaturated fatty acid oleic acid was the major fatty acid (32.6%). The proportions of saturated (34.7%) and polyunsaturated (28.1%) fatty acids were nearly equal.

**Table 3:** The fatty acid composition of the total milk lipids of the mothers (n = 42)

**Fatty acid**	**Common name**	**Mean (SD)**
14:0	Myristic	3.95 (2.10)
14:1	Myristoleic	0.16 (0.09)
15:0	Pentadecanoic	0.36 (0.18)
16:0	Palmitic	23.9 (4.73)
16:1n-7	Palmitoleic	2.38 (0.77)
18:0	Stearic	6.51 (2.18)
18:1n-9	Oleic	32.6 (3.29)
18:1n-7	Vaccenic	2.17 (0.61)
18:2n-6	Linoleic (LA)	22.8 (4.70)
18:3n-6	γ-linolenic	0.23 (0.15)
18:3n-3	α-linolenic (ALA)	1.46 (0.54)
20:2n-6	Eicosadienoic	0.73 (0.28)
20:3n-6	Dihomo-γ-linolenic	0.57 (0.22)
20:4n-6	Arachidonic (AA)	0.93 (0.46)
20:3n-3	n-3 eicosatrienoic	0.10 (0.06)
20:5n-3	Eicosapentaenoic (EPA)	0.26 (0.18)
22:6n-3	Docosahexaenoic (DHA)	0.98 (0.55)
22:1	Erucic	0.35 (0.25)

SD, Standard deviation.

### Comparison of and correlations between the fatty acid compositions of maternal and cord blood phospholipids

We were interested not just in the absolute proportions of particular fatty acids in maternal and fetal (cord) plasma phospholipids, but also in the relative proportions of these fatty acids in the two different plasma samples. Our primary concern was for the long-chain n-3 fatty acids DHA and EPA, the essential fatty acids LA and ALA, and AA. As shown in Table 4, the mean DHA percentage for the mothers’ plasma phospholipids was 5.16%, which is in the middle of the range for most healthy, well-nourished women in other parts of the world. The proportion of DHA in the plasma phospholipids of the newborns’ was about 20% higher compared to their mothers’ mean DHA values. On the other hand, the EPA percentage of the plasma phospholipid fraction of the infants (0.4%) was significantly lower than that of their mothers (0.6%) (p = 0.003). Noteworthy, too, was the finding that the percentages of LA and ALA were approximately 60% and 50%, respectively, lower in the cord plasma phospholipids compared to maternal plasma phospholipids. Equally remarkable was the finding that the percentage of AA, which is one of the two most abundant fatty acids in neonatal brain [31], was about 2-fold higher in the cord plasma phospholipids relative to the maternal phospholipids.

**Table 4:** The fatty acid composition of the plasma phospholipids of the mothers and their newborns

**Fatty acid**	**Common name**	** Mean ± SD**		**P value**
		Mothers (n = 42)	Newborns (n = 42)	
14:0	Myristic	0.46 (0.28)	0.32 (0.12)	0.003
15:0	Pentadecanoic	0.18 (0.05)	0.19 (0.07)	NS
16:0	Palmitic	31.8 (1.61)	31.0 (1.30)	0.017
16:1n-7	Palmitoleic	0.74 (0.34)	0.99 (0.39)	0.003
18:0	Stearic	9.57 (2.03)	12.0 (2.91)	< 0.001
18:1n-9	Oleic	7.75 (1.48)	7.07 (1.03)	0.017
18:1n-7	Vaccenic	1.35 (0.20)	2.48 (0.33)	< 0.001
18:2n-6	Linoleic (LA)	27.7 (3.18)	11.3 (2.97)	< 0.001
18:3n-6	γ-linolenic	0.09 (0.04)	0.20 (0.14)	< 0.001
18:3n-3	α-linolenic (ALA)	0.33 (0.12)	0.17 (0.10)	< 0.001
20:0	Arachidic	0.11 (0.06)	0.14 (0.05)	0.004
20:2n-6	Eicosadienoic	0.45 (0.33)	0.48 (0.68)	NS
20:3n-6	Dihomo-γ-linolenic	2.87 (0.75)	5.47 (0.81)	< 0.001
20:4n-6	Arachidonic (AA)	9.99 (1.97)	19.7 (2.55)	< 0.001
20:5n-3	Eicosapentaenoic (EPA)	0.60 (0.34)	0.40 (0.22)	0.003
22:4n-6	Adrenic	0.36 (0.13)	0.75 (0.52)	< 0.001
22:5n-3	Docosapentaenoic	0.63 (0.19)	0.74 (0.72)	NS
22:6n-3	Ddocosahexaenoic (DHA)	5.16 (1.21)	6.36 (1.81)	0.001

### Correlations

Relationships between the demographic and dietary habits of these same women and the nutritionally-critical n-3 and n-6 fatty acids LA, ALA, AA, EPA and DHA in maternal and neonatal plasma phospholipids and milk are summarized in Table 5. The seafood intake of the mothers was positively and significantly related to the proportion of EPA in the plasma phospholipids of maternal blood (r = 0.51, p < 0.05) and cord blood (r = 0.51, p < 0.01) (Table 5) and the proportion of DHA in breast milk (r = 0.35, p < 0.05) (Table 5). The proportion of LA in the maternal phospholipid fraction was inversely correlated with the parity (r = -0.32, p < 0.05) (Table 5) of the mothers while the ALA percentage in breast milk was correlated negatively with gestational weeks (r = -0.31, p < 0.05) (Table 5).

**Table 5:** Correlations between maternal characteristics and key fatty acids

	**LA**	**ALA**	**EPA**	**DHA**
Gestational week	-	-0.31*^3^	-	-
Parity	-0.32*^1^	-	-	-
Seafood intake	-	-	+0.51*^1^	+0.35*^3^
			+0.51**^2^	

* p < 0.05 ** p < 0.01.

^1^: Maternal plasma phospholipids.

^2^: Neonatal plasma phospholipids.

^3^: Breast milk.

We tested for correlations between the percentages of certain fatty acids between breast milk and the maternal plasma phospholipids (Table 6) and between plasma phospholipids of mothers and babies (Table 7). Statistically significant and positive relationships were found for the proportions of LA, EPA and DHA between breast milk and maternal plasma phospholipids (Table 6). Noteworthy was the observation that the fatty acid status of the infants with regard to DHA appeared to depend on the respective nutritional status of the mother (Table 7) (r = 0.46, p < 0.01).

**Table 6:** Correlations of fatty acids between maternal plasma phospholipids and breast milk

**Breast milk**	**LA**	**EPA**	**DHA**
**Mother blood**			
LA	+0.35*	-	-
ALA	-	-	-
AA	-	-	-
EPA	-	+0.38*	-
DHA	-	+0.35*	+0.43**

* p < 0.05 ** p < 0.01.

**Table 7:** Correlations of fatty acids between maternal and infant plasma phospholipids

**Cord blood**	**LA**	**AA**	**EPA**	**DHA**
**Mother blood**				
LA	+0.38*	-	-	-
ALA	+0.36*	-	+0.40**	-
AA	-	+0.40**	-	+0.36*
EPA	-	-	+0.60**	-
DHA	-	-	+0.55**	+0.46**

*p < 0.05 **p < 0.01.

We inquired whether women who had higher levels of seafood consumption would have higher percentages of essential fatty acids in their plasma phospholipids and milk. However, we did not find any statistically significant relations between the percentages of any fatty acid and the frequency of seafood intake (data not shown).

## Discussion

Docosahexaenoic acid accumulates in neural tissues during fetal and early postnatal development [32,33] and is considered an essential fatty acid for both the fetus and newborn infant [34,35]. It is important for the development of the brain and the retina in mammals, including humans [1,36]. The major determinant of the DHA status of a newborn is the DHA status of its mother. In order to establish and maintain a healthful DHA status during pregnancy and throughout lactation, it is strongly recommended that pregnant and lactating women take in 200-300 mg of DHA per day, either by consuming at least two servings of marine fish per week or by using DHA supplements [5,6].

The motivation behind the present study was our understanding that the DHA status of populations inhabiting islands such as Kinmen in the Taiwan Strait had not been investigated. Consequently, we elected to focus our attention on the question of the DHA status of pregnant women in Kinmen at the time of delivery, and that of their newborns. At the same time we determined the fatty acid composition of the breast milk of these women. The DHA status of an individual can be assessed conveniently and reliably by determining the fatty acid composition of their plasma phospholipids [37]. We have documented herein that the DHA status of pregnant women in Kinmen at the time of delivery appears to be satisfactory, in fact excellent, on the basis of three considerations. First, the mean DHA percentage of the plasma phospholipids of the 42 women was 5.16% (Table 4), which is in the middle of the range of values reported for women elsewhere in the world [38,39]. Second, the mean proportion of DHA in the plasma phospholipids of the cord blood specimens (representing fetal blood) was 6.36% (Table 4), a value which falls at the high end of the range of values reported by investigators in other parts of the world [39-42]. Third, and arguably most importantly from the perspective of the nursing infant, was the exceptionally high DHA proportion (0.98%) we found for the milk fat of the mothers (Table 3). In a review of the literature published in 2007, Brenna and colleagues [30] reviewed data from 25 studies of breast milk fatty acids around the world: the DHA percentage in breast milk ranged from a low of 0.06 (Pakistan) to a high of 0.91 for the Dominican Republic, and the mean DHA percentage for all studies was 0.34. For comparison, the adequate DHA proportion for breast milk in 2008 recommended by the FAO/WHO was between 0.2 and 0.4% of total fatty acids [43]. In a study of maternal DHA supplementation, a saturation point was observed in the relation between the DHA percentage in infants’ red blood cells at 0.8% DHA in breast milk. This saturation phenomenon explains why we did not find any statistically significant correlation between the percentages of DHA in cord-blood serum phospholipids and maternal serum phospholipids.

There is no evidence that DHA percentages above 0.8% in breast milk has any adverse effect on the nursing infant.

The average AA content of the maternal plasma phospholipids of the Kinmen women was 9.99% (Table 4), which is mid-range for corresponding published values; however, at 19.7%, the proportion of AA in their cord plasma phospholipids was above the range of published values for newborns elsewhere in the world which vary from 8.75 to 16.8% [40-42]. Since AA is regarded as a proinflammatory fatty acid, such a large contribution of AA to the fatty acid total in the phospholipids of the newborns should be grounds for concern and perhaps further investigation as well.

It is widely accepted that maternal fatty acid status influences the fatty acid composition of breast milk [44,45]. As has been documented by other investigators [6], the DHA percentage in the milk of the women in the present study was positively correlated with maternal DHA status, as assessed by the DHA percentage for the mothers’ plasma phospholipids (Table 6). The same positive relationship between the proportions of LA and EPA was found between maternal plasma phospholipids and breast milk (Table 6).

The proportions of LA, ALA and EPA were lower in cord plasma phospholipids than in the mothers’ plasma (Table 4). However, the AA and dihomo-γ-linolenic acid percentages were 2-fold higher in maternal plasma compared to cord plasma. Lacking information regarding the oxidation and interconversion of fatty acids in the placenta and fetus or about the transport of fatty acids across the placenta, it is unclear what is responsible for differences we and other have observed in the proportion of particular fatty acids in fetal and maternal blood.

The present study had several limitations. First, since we measured the DHA content of maternal plasma phospholipids only at the time of delivery, the absence of baseline data for the mothers before they became pregnant prevents us from commenting about whether the high DHA status of the participants in the present study in Kinmen were due to their high intake of seafood just during pregnancy or to their intrinsic, normal DHA levels prior to pregnancy. Second, the small sample size in our study limits the generalizability of our findings to all women in Kinmen or other islands in the Taiwan Straits. Furthermore, in recent years there has been an influx into Kinmen of families with pregnant women who are not indigenous to this island. These immigrants may have dietary habits that differ substantially from those of the local Taiwanese population of Kinmen. For example, there is a Chinese custom of ‘doing-the-month’ during the early postpartum weeks when lactating mothers consume large amounts of chicken soup flavored with sesame oil and rice wine. Information regarding similarities and differences of dietary habits during the breastfeeding period between Taiwanese mothers and foreign mothers participating in our study was not obtained.

In light of the very high proportion of DHA we documented in the milk of the Kinmen women (0.98%), it was surprising to find that the average frequency of seafood intake by these women was only 1.6 seafood meals per week. We would expect to have found a higher intake of DHA-rich seafood. A more thorough dietary survey is needed to account for the remarkably high DHA content of the milk of the Kinmen women. Such a study should include a careful survey of the kinds of seafood they consume and the fatty acid composition of those seafood species. Since our study had a cross-sectional design and was limited to analysis of the fatty acid status of lactating women and their newborns, a more comprehensive, long-term longitudinal study would be required to assess the impact of the high DHA content of the milk of Kinmen women on the mental and psychomotor development of their school-aged children. Makrides and colleagues (2010) suggested that the low cognitive level (IQ less than 85) of 60% of slow-developing children could have been avoided if their mothers had consumed adequate amounts of DHA during pregnancy and throughout lactation [46]. The public health implication of such reasoning is that if pregnant women have adequate nutrition with respect to DHA, the incidence of intellectual deficits in their offspring could be reduced. Future follow-up investigations of the newborns in our study are anticipated so that we might correlate the growth and Intelligent Quotient of these individuals with their DHA status as well as with the DHA content of their mothers' milk and the DHA status of their mothers.

It is therefore important for medical personnel and public health officials to educate pregnant women in particular and women of child-bearing age in general of the criticality of a healthful DHA status for themselves and their progeny and to the role seafood can play in this regard.

Finally, the information in the present study should be useful to public health workers in Taiwan in particular who wish to increase the rate of breastfeeding in the nation. As of 2008, only 25.8% of mothers breastfed for at least three months [47] compared to 52%-66% of mothers in most developed countries who breastfeed for at least three months [48]. The exceptionally high DHA content of the milk of Taiwanese women provides an evidence-based argument for promoting breastfeeding in Taiwan.

## Competing interests

The authors declare that they have no competing interests.

## Authors’ contributions

HLH, LTC and RHG proposed the study design and helped to draft the manuscript. LTC performed fatty acid analysis. HHL and CPL participated in data collection. HLH performed statistical analysis. All authors read and approved the final manuscript.
